# Next generation sequencing based assessment of the alloreactive T cell receptor repertoire in kidney transplant patients during rejection: a prospective cohort study

**DOI:** 10.1186/s12882-019-1541-5

**Published:** 2019-09-02

**Authors:** Constantin Aschauer, Kira Jelencsics, Karin Hu, Andreas Heinzel, Julia Vetter, Thomas Fraunhofer, Susanne Schaller, Stephan Winkler, Lisabeth Pimenov, Guido A. Gualdoni, Michael Eder, Alexander Kainz, Heinz Regele, Roman Reindl-Schwaighofer, Rainer Oberbauer

**Affiliations:** 1 Division of Nephrology and Dialysis, Department of Medicine III, Medical University of Vienna, Waehringer Guertel 18-20, 1090 Vienna, Austria; 2 Bioinformatics Research Group, University of Applied Sciences Upper Austria, Softwarepark 13, 4232 Hagenberg im Muehlkreis, Austria; 3 Department of Pathology, Medical University of Vienna, Waehringer Guertel 18-20, 1090 Vienna, Austria

**Keywords:** T cell receptor, Alloreactivity, Kidney transplant, Rejection, Next generation sequencing

## Abstract

**Background:**

Kidney transplantation is the optimal treatment in end stage renal disease but the allograft survival is still hampered by immune reactions against the allograft. This process is driven by the recognition of allogenic antigens presented to T-cells and their unique T-cell receptor (TCR) via the major histocompatibility complex (MHC), which triggers a complex immune response potentially leading to graft injury. Although the immune system and kidney transplantation have been studied extensively, the subtlety of alloreactive immune responses has impeded sensitive detection at an early stage.

Next generation sequencing of the TCR enables us to monitor alloreactive T-cell populations and might thus allow the detection of early rejection events.

**Methods/design:**

This is a prospective cohort study designed to sequentially evaluate the alloreactive T cell repertoire after kidney transplantation. The TCR repertoire of patients who developed biopsy confirmed acute T cell mediated rejection (TCMR) will be compared to patients without rejection.

To track the alloreactive subsets we will perform a mixed lymphocyte reaction between kidney donor and recipient before transplantation and define the alloreactive TCR repertoire by next generation sequencing of the complementary determining region 3 (CDR3) of the T cell receptor beta chain.

After initial clonotype assembly from sequencing reads, TCR repertoire diversity and clonal expansion of T cells of kidney transplant recipients in periphery and kidney biopsy will be analyzed for changes after transplantation, during, prior or after a rejection.

The goal of this study is to describe changes of overall T cell repertoire diversity, clonality in kidney transplant recipients, define and track alloreactive T cells in the posttransplant course and decipher patterns of expanded alloreactive T cells in acute cellular rejection to find an alternative monitoring to invasive and delayed diagnostic procedures.

**Discussion:**

Changes of the T cell repertoire and tracking of alloreactive T cell clones after combined bone marrow and kidney transplant has proven to be of potential use to monitor the donor directed alloresponse. The dynamics of the donor specific T cells in regular kidney transplant recipients in rejection still rests elusive and can give further insights in human alloresponse.

**Trial registration:**

Clinicaltrials.gov: NCT03422224, registered February 5th 2018.

**Electronic supplementary material:**

The online version of this article (10.1186/s12882-019-1541-5) contains supplementary material, which is available to authorized users.

## Background

After the introduction of highly potent immunosuppressive regimens in combination with induction therapy, a significant reduction of acute rejection episodes and an improvement in short term graft survival have been achieved [1, 2]. However, a number of transplanted organs are persistently lost following an acute rejection episode, and long-term graft function and survival following this kind of immunological event poses even greater problems to patient management [3, 4].

Acute cellular rejection with infiltrating CD8+ and CD4+ T lymphocytes play a central role and account for the larger fraction of acute rejection episodes [5]. Lymphocyte infiltrates in the kidney are the leading finding of T cell mediated rejection and therefore the nature of infiltrating lymphocytes in the transplanted kidney is of special interest even more as little is known about the antigen specificity of the T cell receptors and the molecular properties of these infiltrates.

Likewise, T cells play a crucial role in the initiation of antidonor antibody formation and consecutively antibody mediated rejection (ABMR). T helper cells support B cells after encountering their antigen by direct interaction and cytokine production which leads to proliferation, differentiation and affinity maturation of B cells to memory B cells or long-lived plasma cells secreting high-affinity antibodies [6–8].

During early T cell development T cell precursors enter the thymus and differentiate to immature T cells. In the thymus, random recombination of germline-encoded TCR variable (V), diversity (D), and joining (J) gene segments aims for the formation of a functional TCR protein and generation of a highly diverse TCR repertoire [9].

Junctional diversification further increases the combinatorial diversity by trimming gene ends or adding nucleotides between the recombining genes and thereby generating hypervariable complementarity-determining regions (CDR1-CDR3). The complementarity-determining region 3 (CDR3), which is the most variable CDR, spans over the V(D) J junction and forms the primary site of antigen contact [9, 10]. The cells expressing a recombined T cell receptor only survive if self MHC (major histocompatibility complex) and a self-peptide presented by thymic antigen presenting cells is recognized with adequate affinity to transmit a survival signal (positive selection). In a second selection process (negative selection) T cells with an operational TCR but an activation above an affinity- or cross-reactivity threshold are send to apoptosis by a death signal as these cells would be more likely to become activated by self-peptides or by the MHC molecule itself and cause autoimmunity [9].

This complex development of the T cell receptor repertoire and selection process results in the generation of a highly diverse repertoire capable of defending an individual against a variety of pathogens and being ideally tolerant to antigens present in the own body. This leads to a large number of unique TCR rearrangements with in theory over 10^13 unique T cell clonotypes representing the immune repertoire [11–13].

After the generation of the immune repertoire events such as infections, aging or immune modulating therapies cause an alteration which can lead to the expansion or deletion of clones and effect the diversity of the overall repertoire [10, 14, 15].

In solid organ transplantation T cells recognizing a foreign antigen or MHC protein, so called alloreactive T cells, expand and cause a rejection with severe organ damage. Receptor diversity analysis of these alloreactive cells using conventional methods such as PCR-based hybridization methods, flow cytometry of V region usage or TCR spectratyping, is a major technical challenge [16–18]. Additionally, these techniques have limitations regarding their resolution:

Fluorescence activated cell sorting (FACS) is limited by the availability of antibodies against T cell receptor’s Variable, Diversity and Joining (VDJ) elements and PCR-based length analysis by spectratyping does not allow to discriminate between clones with identical CDR3 lengths [19, 20].

With the development and technical progress of next-generation sequencing (NGS) technologies it is now feasible to sequence millions of receptor clones simultaneously, which provides a much more detailed and precise picture of the immune repertoire [11, 21, 22]. It is possible to obtain several million sequences per sequencing run and only recently the focus has been put on receptor sequencing of the alloreactive lymphocyte population and the changes in abundancy of these clones and repertoire diversity over time [23].

Two studies of TCR repertoire analysis based on NGS data were published in 2009 using two different experimental designs [11, 21]. These designs differ in the starting material used being either genomic DNA (gDNA) or messenger RNA/complementary DNA (mRNA/cDNA)-based approaches [24, 25]. Both strategies have advantages but also shortcomings. Using genomic DNA in contrast to mRNA as a starting template has the advantage of superior quantification as every T cell only possesses one functional TCR chain, depicting in theory a more precise overall T cell receptor repertoire. A major downside of using gDNA is the significant multiplex PCR bias introduced by different primer annealing or amplification efficiencies leading to over/underrepresentation or dropout of those elements by non-uniform amplification. This bias is reduced in the mRNA/cDNA approach by using template switch during cDNA library preparation which forms universal primer annealing sites on both ends of the library and can be further counteracted by the introduction of unique molecular identifiers (UMIs). However, the mRNA/cDNA approach is known to be less specific and conclusions on absolute frequencies of cells is more difficult due to unequal mRNA expression levels in cells.

Morris et al. recently used mixed lymphocyte reactions (MLR, incubating donor antigen presenting cells (APCs) with recipient T cells) to select alloreactive T cell clones prior to transplantation. Using high-throughput sequencing data of the CDR3 a unique fingerprint of the donor-reactive T cell repertoire was identified before transplantation and donor reactive T cells were tracked posttransplant in blood samples [26]. Further studies validated this approach in different solid organ settings, as in small bowel transplantation, also analyzing T cell repertoires in tissue biopsies [27, 28].

We aim to apply a similar approach in this project to get a closer insight into the alloreactive lymphocyte population prior to transplantation and follow these populations over time and analyze associations of repertoire diversities with the histomorphological phenotype of T cell mediated rejection in the kidney transplantation setting. Further we would like to decipher patterns of expansion of these clones prior to rejection and decline in graft function to hopefully foresee rejection episodes before graft damage occurs.

## Methods/design

This is a single center prospective cohort trial designed to evaluate the value of T cell receptor repertoire sequencing of T cells in the periphery and in the allograft during a biopsy proven rejection episode compared to absence of histological findings of rejection conducted at the General Hospital of Vienna/Medical University of Vienna, Department of Nephrology.

These alloreactive T cells are defined prior to transplantation via a mixed lymphocyte reaction as shown in previous studies [26, 27].

Fifteen patients with histological proven acute T cell mediated rejection will be compared to 15 patients without histopathological signs of alloimmune response in time matched for-cause biopsies. Only recipients of donor organs evaluated and accepted by the local transplant team in Vienna will be included due to lack of availability of donor peripheral blood mononuclear cells (PBMCs) of donors evaluated by Eurotransplant, an Europe-wide program allocating and exchanging deceased donor organs across-borders [29]. The proposed duration of the trial is 36 months. The sample size of 15 patients experiencing an acute T cell mediated rejection and an accrual phase of 24 months was calculated based on historic data from our center. The study protocol and informed consent were designed following the “Standard Protocol Items: Recommendations for Interventional Trials (SPIRIT) 2013 statement” guidelines and the standards of the world health organization research ethics review committee (Additional files 1 and 2) [30, 31].

### Outcome parameters

Expansion or deletion of the pre-transplant defined alloreactive T cell repertoire against the allograft during acute T cell mediated rejection will be compared to patients without biopsy proven rejection. Further analysis of local T cell expansion in the allograft during a rejection episode will be performed via RNA extraction of the frozen biopsy. Additionally an analysis will concentrate on T cell clonality analysis and repertoire diversity of patients over time and of rejecting and non-rejecting individuals.

### Sample collection

**Fig. 1:**
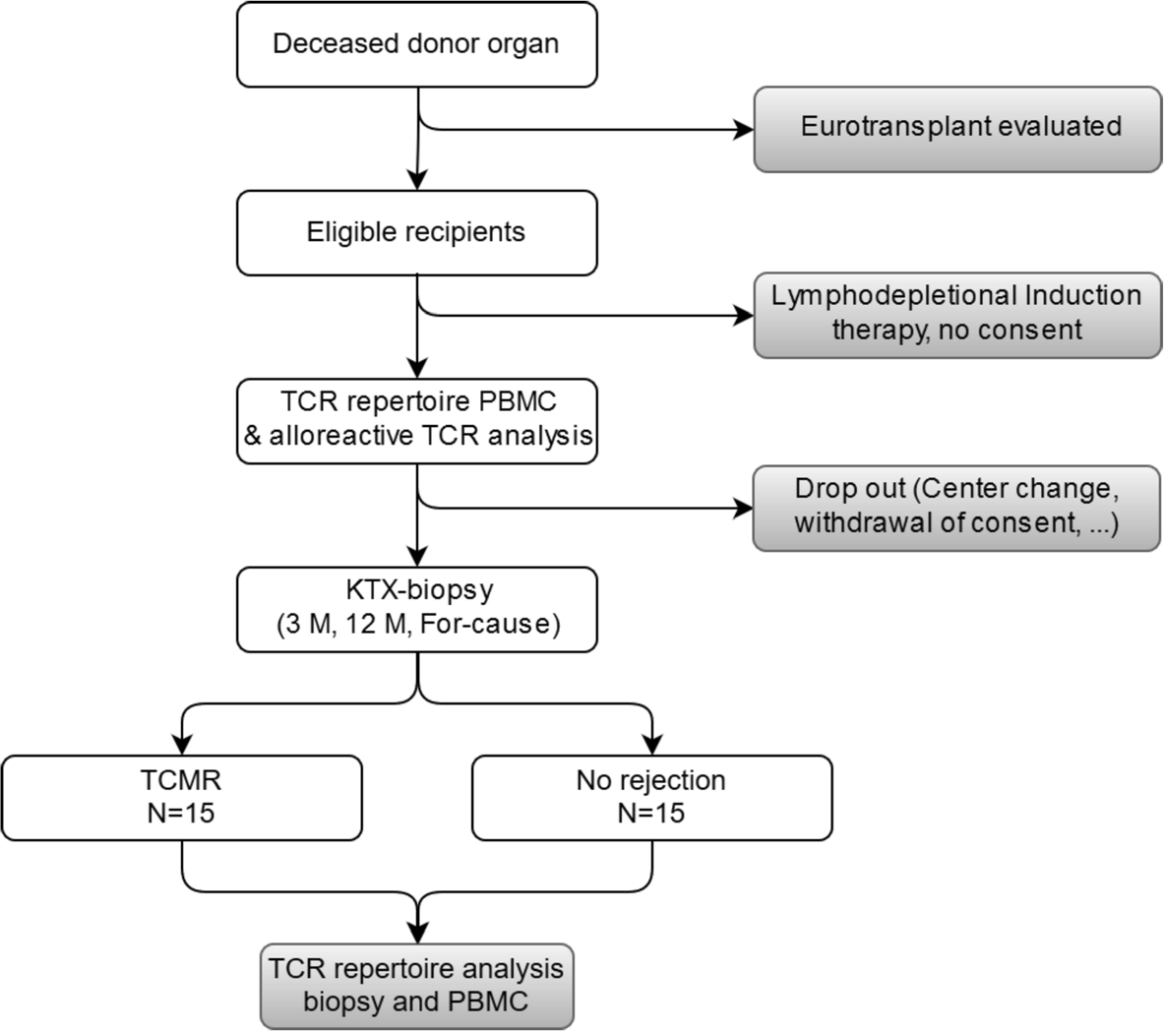
Study Flowchart. All eligible patients receiving a kidney transplant from donors evaluated at our center will be included and TCR repertoire of total PBMCs and alloreactive T cells will be analyzed. TCR repertoire in the periphery and in biopsies will be assessed at surveillance biopsies and TCMR. DSA: donor reactive antibodies, TCR: T cell receptor, PBMC: peripheral blood mononuclear cells, KTX: kidney transplant, TCMR: T cell mediated rejection

For our analysis we will include a minimum of 150 kidney transplant recipients with a for-cause biopsy after transplantation that were enrolled prospectively between february 2018 and february 2020. A medical doctor obtained informed consent of eligible recipients prior to transplantation and recipient and donor PBMCs will be stored at time of transplantation. Only recipients of donors evaluated by the local transplant coordinators will be included in this study due to availability of donor PBMCs. Additionally, surveillance biopsies will be performed at 3 and 12 months and tissue samples as well as PBMCs will be stored in liquid nitrogen and − 80 °C (Fig. 1). De-identification of included patient samples is performed before sample processing by a study specific patient identifier.

**Fig. 2:**
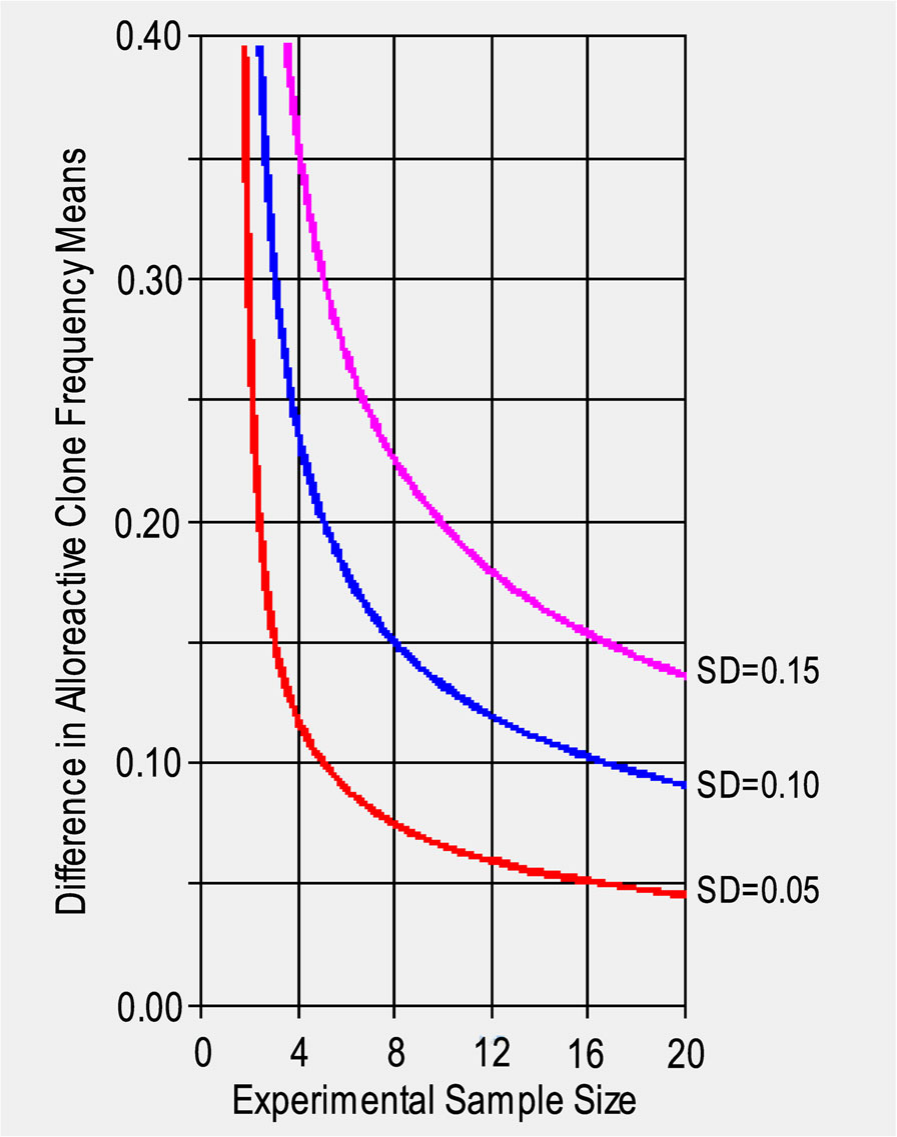
Detectable effect size versus sample size. Depicted is the relationship between number of samples in the study and minimal detectable difference in population means for standard deviations of 0.05 (red), 0.1 (blue) and 0.15 (pink). SD: Standard deviation

Including 15 samples per group (Rejectors, non-rejectors) our study has 80% power to detect a minimum difference of 0.106 in the mean proportion of alloreactive clones in the posttransplant repertoire of patients from the two groups assuming a standard deviation of 0.1 each. This calculation was performed using Power and Sample Size Version 3.0 (Fig. 2) [32].

### Inclusion and exclusion criteria

Inclusion criteria for this study are: (1) Kidney transplant recipient, (2) age ≥ 18 years, (3) donor evaluation by the local transplant coordinators and (3) written informed consent. Exclusion criteria for this study are: (1) Donor evaluation by Eurotransplant or (2) lymphodepletional induction therapy.

### Blood samples

At time of transplantation and at time of biopsies as well as 3 and 12 months after transplantation, blood samples will be collected as part of the routine blood draw at the follow up at the outpatient clinic (Table 1). PBMC isolation will be carried out as described.

**Table 1:** Schematic diagram of the patient’s timeline (adjusted from SPIRIT guidelines for trial protocols)

Timepoint	Study period				
	Enrolment	Biopsy			Close-out
	Transplantation	For cause	M3	M12	M24
Enrolment:					
Eligibility screen	X				
Informed consent	X				
Interventions:					
PBMC collection	X	X	X	X	
Kidney Biopsy		X	X	X	
Assessments:					
TCR repertoire sequencing of unstimulated PBMCs	X	X	X	X	
TCR sequencing of Alloreactive T cells after MLR	X				
Phenotypic analysis of peripheral T cell populations	X	X	X	X	

*M3* Month 3, *M12* Month 12, *MLR* Mixed lymphocyte reaction, *TCR* T cell receptor, *PBMC* Peripheral blood mononuclear cells

### PBMCs isolation

PBMCs of donors evaluated by our transplant center at the Medical University of Vienna will be isolated via density gradient from whole blood which will be sent along with the renal allograft at transplantation. Recipient PBMCs will be isolated out of whole blood via density gradient at time of transplantation, as described previously [26, 27].

After isolation cells will be stored in liquid nitrogen and − 80 °C for future analysis.

### Tissue samples

#### Surveillance biopsies

At 3 and 12 months surveillance biopsies are performed at our transplant center for patients included in the study with informed consent prior to transplantation and tissue as well as blood samples will be stored.

All biopsies will be evaluated on standard formalin fixed paraffin-embedded (FFPE) sections at the Department of Pathology as part of the clinical routine. Histological slides from FFPE tissue will be made and will be used for routine staining (Hematoxylin-Eosin, Acid Fuchsin Orange G (AFOG), Periodic acid Schiff, Methenamine) and immunohistochemistry. Histopathology scoring will be performed according to BANFF 2017 [33].

A second core will be stored at − 80 °C (in tissue plus OCT compound) until DNA and RNA will be extracted for further analysis (see below).

#### For-cause biopsies

For-cause biopsies are performed routinely in patients with delayed or deteriorating graft function with a low diagnostic threshold to guide and optimize clinical decision-making. The evaluation and biopsy collection will be performed as mentioned above.

### In vitro expansion of donor-specific alloreactive T cell lymphocytes

The protocol of in vitro expansion of donor-specific T cell lymphocytes will be performed as described previously [26]. In short, we will label donor cells with BD HorizonTM Violet Proliferation Dye 450 (VPD-450 catalog # 562158) and recipient cells with carboxyfluorescein succinimidyl ester (CFSE) according to the manufacturer’s protocol prior to resuspension in MLR medium (AIM-V supplemented with 5% AB heat- inactivated human serum, 0.01 M HEPES, and 50 μM 2-ME and 1% Pen/Strep) at a concentration of 2 × 10^6^ cells/mL. Donor cells will additionally be irradiated at 35 Gray [27].

#### Preparation of CFSE-labeled responder cells

After thawing cryopreserved recipient PBMCs will be labeled with carboxyfluorescein succinimidyl ester (CFSE). Subsequently cells will be washed twice, resuspended in MLR medium at 2 × 10^6^ cells/mL.

MLR will be performed by plating 2 × 10^5^ CFSE-labeled transplant responder cells and 2 × 10^5 VPD-labeled irradiated stimulator cells will be plated in each well of a 96-well plate (total well volume 200 μl). MLR cultures will be incubated at 37 °C for 6 days. After incubation, the different dyes will allow us to differentiate between the cells of interest (CFSE- labeled recipient cells) and the unessential cells (VPD-labeled donor cells) [27].

### Fluorescence activated cell sorting (FACS)

Cells will be harvested from the 6 day MLR culture from the 96 well plate, washed with FACS Buffer and counted. Unstimulated PBMCs will be thawed, washed twice with media and PBS, resuspended in FACS buffer and counted [26, 27]. Subsequently the cells will be resuspended in FACS buffer, blocked for 30 min with blocking buffer (10% human serum, 1% BSA) stained for 30 min with fluorochrome-conjugated anti-CD3, anti-CD4, anti-CD8, anti-CD25, anti-CD127, anti-CD45RA and anti-CCR7. Sorting will be done on a FACS Aria II high speed cell sorter. We will isolate two populations after MLR (CFSE low CD4/CD8) and three discrete cell populations for unstimulated PBMCs (CD4, CD8, Treg). Phenotypic analysis of the unstimulated PBMCs pre-and posttransplant includes the following markers:

T helper cells (CD3+, CD4+, CD8-), cytotoxic T cells (CD3+, CD4-, CD8+), Treg cells (CD4+, CD25+++, CD127-, FOX-P3+), central memory cells (CD4/CD8, CD45RA-, CCR7+), effector memory cells (CD4/CD8, CD45RA-, CCR7-) [27, 34]. The sorting of T cell populations is followed by RNA isolation and library preparation for TCR sequencing.

### T cell receptor repertoire sequencing

#### RNA isolation

RNA isolation is done following the original Trizol protocol (Invitrogen, Carlsbad, CA).

#### NGS library preparation

The library preparation is described in [35]. Total RNA from PBMCs was transcribed using the SmartScribe kit (Clontech, USA) and universal primers specific for the constant region of T cell receptor.

Custom cap-switching oligonucleotides with unique molecular identifiers (UMI) and sample barcodes were used to introduce the universal primer binding site to the 3′ end of the cDNA molecules. cDNA was treated (45 min, 37 °C) with 5 U UDG (NEB, USA) for 30 min, 37 °C, followed by a purification (Qiagen PCR purification kit).

PCR amplification was used to amplify the cDNA and to introduce Illumina TruSeq adapters and a second sample barcode. The first PCR step consists of 16–18 cycles of: 94 °C for 20 s., 60 °C for 15 s., 72 °C for 60 s. Reactions were done in a total volume of 15 μl (1x Q5 polymerase buffer (NEB), 5 pmol of Sm1msq and RPbcj1, RPbcj2, RPacj primers, dNTP (0.125 mM each), 0.15 μl of Q5 polymerase). One microliter of the purified PCR product was used for the second amplification step (94 °C 20 s, 60 °C 15 s, 72 °C 40 s 12–18 cycles).

Total reaction volume of 25 μl: 1x Q5 polymerase buffer, 5 pmol of Smoutmsq and “Il-bcj-ind” or “Il-acj-ind” primers (with sample specific Illumina index sequences), dNTP (0.125 mM each) and 0.25 μl of Q5 polymerase. Size selection and purification for 500-800 bp fragments of the purified PCR product was performed using SPRI bead technology (Beckman Coulter, CA, USA).

A detailed list of Primer sequences used has been published previously [15].

### Bioinformatics

**Fig. 3:**
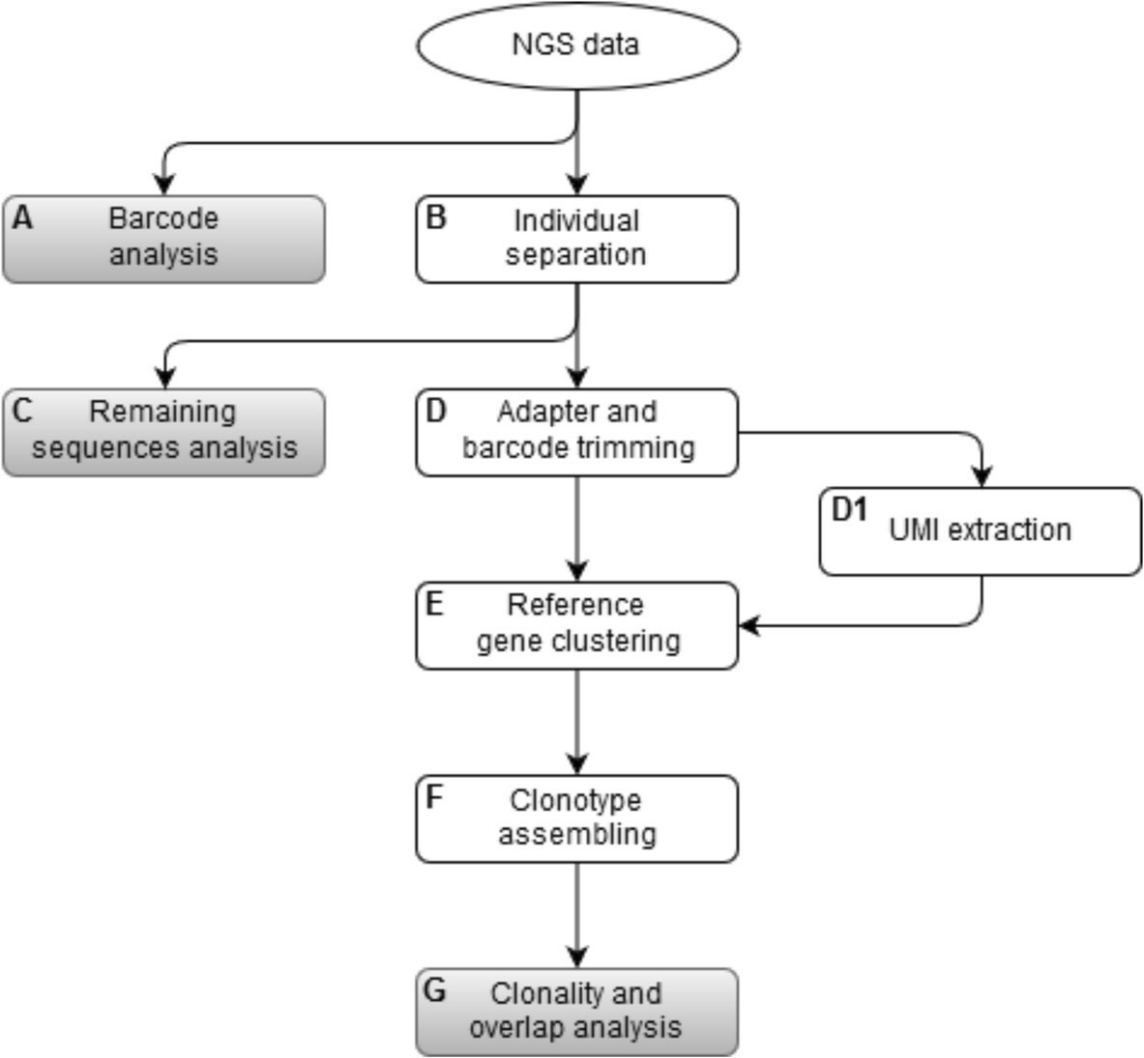
Bioinformatic Flowchart. A pipeline for the analysis of T cell repertoires. A: First, barcode analysis is done as well as separating the sequences belonging to the different individuals (B). C: Reads that cannot be assigned to a specific individual are stored in a separate FASTQ file for later investigation regarding the origin of these sequences. D: Adapters and barcodes from sequences are trimmed. During this step the UMIs are determined and stored in a separate file (D1). E: Afterwards the sequences are clustered with respect to their reference genes and UMIs. F/G: Clonotypes are assembled and clonality, diversity and repertoire overlap analysis is performed

A computational workflow for the evaluation of results from NGS based analysis of T cell receptor repertoires will be established. The workflow will cover all aspects of data quality evaluation, demultiplexing of samples and the determination of clonality and diversity (Fig. 3).

FastQC will be employed for quality control of raw sequencing data. The quality of each run will be further checked by analyzing the frequency of sample barcodes, their position in the reads, as well as the recovery of phiX. Only reads passing the initial quality control will be analyzed further. Sequences assigned to the individuals characterized by unique nucleotide barcodes are identified and separated.

In preparation for determining the immunological interesting regions (especially the V(D) J regions), the appendages will be trimmed to remove barcodes and any adapters. In the same step the UMIs will be extracted and stored separately for subsequent use during reference gene clustering. Remaining sequences which either miss barcodes or have invalid barcodes will be collected for further analysis of the origin of these sequences.

After preprocessing V(D) J region containing reads will be subjected to reference gene clustering. Therefore, all reads will be aligned to T cell receptor reference sequences, using *Bowtie2*, a tool for aligning sequencing reads to reference genes [36]. Subsequently, reads aligned to the same position in a reference sequence and sharing an UMI will be collapsed into a single read.

Finally, ImmunExplorer will be used to perform clonotype assembly based on the reference gene clustered sequences with MiXCR and to carry out clonality, diversity and repertoire overlap analysis [37, 38].

### Statistical methods

After identification of alloreactive T cell clones prior to transplantation-via one way MLR- the proportion of alloreactive clones in the posttransplant repertoire of rejectors and non-rejectors will be compared by means of t-test.

### Study registration

The study was registered in a public clinical trial database on February 5th 2018 (ClinicalTrials.gov NCT03422224) [39].

## Discussion

Adaptive immunity and T cell activation plays a crucial role in rejection of solid organ transplants [40, 41]. Until now the description of T cells responsible for allograft rejection has been elusive mostly due to technical limitations. With the rising of cutting edge techniques as next generation sequencing it has been possible to characterize and track alloreactive T cells potentially responsible for rejection episodes.

In the transplantation setting the questions raised using these novel methods have until now mostly been focusing on elucidating the mechanisms of tolerance induction in the setting of combined kidney and bone marrow transplantation [26]. In the paper of Morris et al., who were one of the first using the technique of TCR sequencing of alloreactive T cells and tracking these over time, showed a decline of these distinct clones in tolerant patients compared to no significant reduction in a patient experiencing a rejection episode.

These findings raise potential questions if a similar pattern of increase of alloreactive clones in the periphery can be seen in kidney transplant patients at a rejection episode. Of additional interest in our study will be if these alloreactive clones expand and can not only be tracked in the periphery but also in the allograft itself during rejection. These questions have partially been raised in two papers published by two different groups [42, 43].

The results were promising although patient numbers with complete follow up in the paper of Alachkar et al. were low and emphasis was not put on alloreactive clones, but more on TCR clone kinetics during rejection [43]. The detection and TCR sequencing of the pre-transplant defined alloreactive T cell clones targeting the allograft and causing rejection episodes may open new opportunities to predict rejections prior to clinical deterioration of graft function. Further the identification of the patients individual alloreactive T cell repertoire can potentially help to decipher antigen specificity of these cells as new techniques show promising results in predicting epitopes targeted by T cells [44, 45].

The antigen-prediction system developed in the paper of Glanville et al. managed to assign TCRs to antigen-specific binding groups and define enriched sequence motifs which were mainly responsible for antigen binding. They used these motifs to design a set of synthetic TCRs not found in the biological samples and predict their specificity to a known antigen. These synthetic TCRs were proven to successfully induce T cell activation upon antigen contact.

By using the TCR repertoire sequencing approach it will generally be possible to identify T cell clonotypes which are expanded compared to the baseline repertoire and decipher changes in overall diversity and clonality. The analysis of the expanded T cell population taking the clinical phenotype of a patient into consideration could provide new insights into several questions. In delicate situations for example as rise in serum creatinine in presence of a coexisting significant viraemia (e.g. BK Virus, Cytomegalovirus), an expansion of a virus specific T cell population compared to alloreactive clones could give important information for clinical decision making, as virus specific T cells have already been isolated and sequenced successfully [42, 46–50]. Zeng et al. compared the frequency of BK virus and alloreactive T cell clones, defined by flow cytometric measurement of T cell responses to HLA and BK peptide stimulation in vitro, in the periphery and in kidney biopsies of transplant patients during BK nephropathy and rejection [42]. They found that biopsies with BK nephropathy contain more alloreactive than virus specific clones and theorize that the leading element of tissue injury in viral nephropathy is primary mediated by an ‘innocent bystander’ mechanism and secondary T cell migration caused by both anti-viral and anti-HLA immunity.

Dziubianau et al. included next to BK virus also CMV specific T cell clones in their analysis but final conclusions were hampered by the small sample number [46]. Nevertheless, they could prove TCR repertoire sequencing as a reliable and reproducible technique for the detection of T cell mediated pathology.

Another possible use could be the description of T cell plasticity regarding the conversion of pre-transplant non Treg, effector/central memory cells into Tregs, or other effector cells which were defined by their distinct T cell receptor. This has been already partly shown in a paper of Sprangers et al. [27].

Besides T cell conversion also T cell reconstitution after anti-thymoglobulin (ATG) induction has been studied by several research groups [51–55]. The results showed an imbalanced reconstitution of the entire T cell population and an altered response of alloreactive T cells after ATG induction therapy [51, 56]. But there was no specific emphasis put on the reconstitution of the alloreactive T cell population.

Regarding the Treg subpopulation, also the properties of specific Treg cells defined by their TCR in tolerant patients or patients with good graft function and low amount of immunosuppressant therapy, compared to patients experiencing rejection episodes as well as the influence of immunosuppressants (e.g calcineurin inhibitors), which are known to particularly affect this population, could be of interest [57, 58].

This promising technique shows to have a broad field of application giving answers to diverse questions regarding the T cell repertoire in kidney transplantation.

An analysis of our data repository showed that these 30 samples will be available within the first 2 years after study initiation accounting for a transplant frequency of local evaluated donors of roughly 120 per year at our center.

Only biopsy proven T cell mediated rejections according to current pathology classification, BANFF 2017 or total absence of rejections will be included in our analysis.

A shortcoming of this study will be a technical limitation regarding the definition of alloreactive T cell clones as the MLR mostly represents antigen presentation via the direct pathway and only to a fewer extent the indirect pathway, which seems the leading mechanism behind chronic allograft function. This may be limiting or hamper conclusions made to chronic allograft nephropathy but should not compromise the assertions concluded in the setting of acute T cell mediated rejection in the first months of transplantation.

## Additional files

Additional file 1: Informed consent Version 1 for the research study: Next generation sequencing based assessment of the alloreactive T- cell receptor repertoire in kidney transplant patients during rejection: a prospective cohort study. (PDF 158 kb)

Additional file 2: EQUATOR Network Reporting Checklist. “Standard Protocol Items: Recommendations for Interventional Trials 2013 statement” checklist. (PDF 76 kb)

## Data Availability

Patient information will be stored at the main data repository of the general hospital of Vienna, following international standards of patient data security in accordance with the Austrian Data Protection Act. Biological samples will be stored at the Biobank of the General Hospital/Medical University of Vienna, where samples will be labeled with specific patients’ identifier, unique for the biobank. The decrypting code is separated strictly from the encoded records and only the person in charge of this project will be able to link personal data (name, sex) and genetical information (CDR3 region of the TCR). Access to clinical data and biological samples will be limited to authorized persons. For this study no data monitoring committee (DMC) will be installed as this is a sponsor independent non interventional single center study [59]. The datasets used and analyzed during the current study are available from the corresponding author on reasonable request.

## Abbreviations

ABMR: Antibody mediated rejection
AFOG: Acid fuchsin orange G
APC: Antigen presenting cell
ATG: Antithymocyte globulin
cDNA: Complementary DNA
CDR3: Complementarity determining region 3
CFSE: Carboxyfluorescein succinimidyl ester
FACS: Fluorescence activated cell sorting
FFPE: Formalin fixed paraffin-embedded
gDNA: Genomic DNA
IMEX: ImmunExplorer
IMGT: ImMunoGeneTics
MHC: Major histocompatibility complex
MLR: Mixed lymphocyte reaction
mRNA: Messenger RNA
NGS: Next-generation sequencing
PBMC: Peripheral blood mononuclear cell
SPIRIT: Standard protocol items: Recommendations for interventional trials statement
TCMR: T cell mediated rejection
TCR: T cell receptor
UMI: Unique molecular identifiers
V(D)J: Variable, Diversity, Joining
VPD-450: Violet Proliferation Dye 450
