# Supplementary material for: Next generation sequencing based assessment of the alloreactive T cell receptor repertoire in kidney transplant patients during rejection: a prospective cohort study
Source: BMC Nephrol. 2019 Sep 2;20:346. doi: 10.1186/s12882-019-1541-5 (PMC6719356; doi:10.1186/s12882-019-1541-5)
Supplement: Supplementary file 1 — Informed consent Version 1 for the research study: Next generation sequencing based assessment of the alloreactive T- cell receptor repertoire in kidney transplant patients during rejection: a prospective cohort study. (PDF 158 kb) [file 12882_2019_1541_MOESM1_ESM.pdf]

Informed consent for the research study:

Next generation sequencing based assessment of the alloreactive T- cell receptor repertoire in kidney transplant patients during rejection: a prospective cohort study

Informed Consent form for male and female kidney transplant recipients who undergo a kidney transplantation at the General Hospital Vienna/Medical University of Vienna, and who we are inviting to participate in research on the cellular Immune response responsible for graft rejection. The title of our research project is **"Next generation sequencing based assessment of the alloreactive T- cell receptor repertoire in kidney transplant patients during rejection: a prospective cohort study"**

Principal Investigator:
Ao. Univ. Prof. Rainer Oberbauer, MD/PhD

Investigation site:
Medical University of Vienna

Funding institution:
Austrian National Bank (OeNB)

This Informed Consent Form has two parts:

- **Information Sheet (to share information about the research with you)**
- **Certificate of Consent (for signatures if you agree to take part)**

You will be given a copy of the full Informed Consent Form

PART I: Information Sheet

Introduction

Dear participant!

As you are listed for a potential Kidney Transplantation at our institution, we invite you to participate in the study named above.
The research goal of this study is to increase the survival of kidney transplant recipients and their grafts. You do not have to decide today whether or not you will participate in the research. Before you decide, you can talk to anyone you feel comfortable with about the research.
There may be some words that you do not understand. Please ask me to stop as we go through the information and I will take time to explain. If you have questions later, you can ask them of me, the study doctor or the staff.

Purpose of the research

After a kidney transplantation the patient's immune system has to be controlled to avoid it attacking the transplanted kidney. For this need it is essential for every patient to closely take their immunosuppressive therapy. Nevertheless patients still suffer from rejection episodes and consecutive graft loss, after kidney damage occurred. Nowadays we know that a specific cell population is responsible for recognizing and damaging the graft but very few is known about this population.
The purpose of this study is to identify and describe cells responsible for a rejection episode and graft failure.

Type of Research Intervention

To participate in this study no additional interventions or appointments will be necessary. At the day of transplantation, 4 vials of 9ml whole blood will be taken additionally to the routine blood draw you will have prior to transplantation.
After successful transplantation every patient treated at our center receives a routine management biopsy at three and twelve months after transplantation following international guidelines. At these timepoints, next to the routine blood draw to exclude biopsy related complications, additional 4 vials of 9ml blood will be drawn. Also part of the biopsy taken-if not needed for diagnostic reasons-will be conserved for further analysis.

Participant selection

We are inviting all patients receiving a kidney transplant at our center to participate in this research project.

Voluntary Participation

Your participation in this research is entirely voluntary. It is your choice whether to participate or not. Whether you choose to participate or not, all the services you receive at this clinic will continue and nothing will change. If you choose not to participate in this research project, you will be offered the treatment that is routinely offered in this hospital for kidney transplant recipients, and we will tell you more about it later. You may change your mind later and stop participating even if you agreed earlier.

Procedures and Protocol

After successful blood draw it will be processed and a distinct cell population (PBMC) will be isolated and stored at the biobank facility of our hospital. At transplantation your cells will be mixed and incubated with donor cells in a mixed lymphocyte reaction (MLR), to distinguish the donor reactive cell population. Subsequently these cells will be isolated and genetic analysis will be performed by next

generation sequencing (NGS). During the follow up timepoints this cell population will be followed in the blood and kidney biopsy and recognized by their genetic properties.

Unfamiliar Procedures

Mixed lymphocyte reaction (MLR):
Participant lymphocytes will be incubated with donor cells to distinguish donor directed cells.
Next generation sequencing (NGS):
Recent technology to fast and efficiently sequence parts or whole genome

Treatment:

You will receive the treatment of a kidney transplant recipient according to international guidelines. This means that every kidney transplant recipient will be taking immunosuppression and in case of rejection a small biopsy of your kidney will be taken. This biopsy will be done in accordance with the guidelines saying that the sample must be taken using a local anesthesia which means that we will give you an injection close to the area where we will take the sample from. This will make the area numb so that you will not feel any pain when we take the sample.
4 hours after the biopsy a routine blood draw is performed to verify the absence of bleeding at the biopsy site.

Description of the Process

Duration

The participants will be followed up for 1 year after Transplantation. During that time, you just have to attend your routine appointments in the clinic. No additional visits or interventions will be necessary for the participation at this study.

Side Effects

Possible side effects are not explicitly related to the study protocol as all blood draws or biopsies necessary for this research are part of routine or medically indicated procedures.

Benefits

There will not be any direct benefit for the study participant but your participation is likely to help us find the answer to the research question. There may not be any benefit to the society at this stage of the research, but future generations are likely to benefit.

Reimbursements

There will be no financial incentives or reimbursement as the participants will not have additional visits and no supplemental costs or time loss due to this study

Confidentiality

The information that we collect from this research project will be kept confidential. Information about you that will be collected during the research will be put away and no-one but the researchers will be able to see it. Any information about you will have a number on it instead of your name. Only the researchers will know what your number is and we will lock that information up with a lock and key. It will not be shared with or given to anyone.

Right to Refuse or Withdraw
You do not have to take part in this research if you do not wish to do so and refusing to participate will not affect your treatment at this clinic in any way. You will still have all the benefits that you would otherwise have at this clinic. You may stop participating in the research at any time that you wish without losing any of your rights as a patient here. Your treatment at this clinic will not be affected in any way.

Who to Contact
If you have any questions you may ask them now or later, even after the study has started. If you wish to ask questions later, you may contact any of the following:

Ao. Prof. Rainer Oberbauer, MD/PhD
rainer.oberbauer@meduniwien.ac.at
01 40400 43900

Dr. med. Constantin Aschauer
Constantin.aschauer@meduniwien.ac.at
01 40400 43890

Dr. med. Roman Reindl-Schweighofer
Roman.reindl-schweighofer@meduniwien.ac.at
01 40400 43910

This proposal has been reviewed and approved by the local ethics committee (EK NB: 1973/2017, signed 7/11/2017), which is a committee whose task it is to make sure that research participants are protected from harm. If you wish to find about more about the IRB, contact: ethik-kom@meduniwien.ac.at.

You can ask me any more questions about any part of the research study, if you wish to. Do you have any questions?

PART II: Certificate of Consent

I have read the foregoing information, or it has been read and explained to me by Dr. __________________ . I have had the opportunity to ask questions about it and any questions that I have asked have been answered to my satisfaction. I consent voluntarily to participate as a participant in this research.

Print Name of Participant__________________

Signature of Participant __________________

Date ___________________________
Day/month/year

If illiterate

I have witnessed the accurate reading of the consent form to the potential participant, and the individual has had the opportunity to ask questions. I confirm that the individual has given consent freely.

Print name of witness_____________________ AND Thumb print of participant

Signature of witness ______________________

Date ________________________
Day/month/year

Statement by the researcher/person taking consent

I have accurately read out the information sheet to the potential participant, and to the best of my ability made sure that the participant understands that the following will be done:

1. Additional 4 vials of 9ml blood at routine blood draws (at Transplantation, rejection, Month 3, Month 12)
2. Parts of the kidney biospy will be taken for research purpose if not needed for diagnosis
3. Genetic analysis will be performed with the tissue and blood collected

I confirm that the participant was given an opportunity to ask questions about the study, and all the questions asked by the participant have been answered correctly and to the best of my ability. I confirm that the individual has not been coerced into giving consent, and the consent has been given freely and voluntarily.

A copy of this ICF has been provided to the participant.

Print Name of Researcher/person taking the consent________________________

Signature of Researcher /person taking the consent__________________________

Date ___________________________
Day/month/year
